# Eating Disorders, Heredity and Environmental Activation: Getting Epigenetic Concepts into Practice

**DOI:** 10.3390/jcm9051332

**Published:** 2020-05-03

**Authors:** Howard Steiger, Linda Booij

**Affiliations:** 1 Eating Disorders Continuum, Douglas University Institute, Montreal, Quebec H4H 1R3, Canada; 2 Douglas Institute Research Centre, McGill University, Montreal, Quebec H4H 1R3, Canada; 3 Department of Psychiatry, McGill University, Montreal, Quebec H3A 1A1, Canada; 4 Department of Psychology, Concordia University, Montreal, Quebec H4B 1R6, Canada; 5 Sainte-Justine Hospital Research Centre, University of Montreal, Montreal, Quebec H3T 1C5, Canada

**Keywords:** epigenetics, anorexia nervosa, bulimia nervosa, eating disorders, DNA methylation, gene-environment interactions

## Abstract

Epigenetic mechanisms are believed to link environmental exposures to alterations in gene expression, and in so doing, to provide a physical substrate for the activation of hereditary potentials by life experiences. In keeping with this idea, accumulating data suggest that epigenetic processes are implicated in eating-disorder (ED) etiology. This paper reviews literature on putative links between epigenetic factors and EDs, and examines ways in which epigenetic programming of gene expression could account for gene-environment interactions acting in the EDs. The paper also presents evidence suggesting that epigenetic processes link malnutrition and life stresses (gestational, perinatal, childhood, and adult) to risk of ED development. Drawing from empirical evidence and clinical experience, we propose that an epigenetically informed understanding of ED etiology can benefit patients, caregivers, and clinicians alike, in the sense that the perspective can reduce judgmental or blameful attitudes on the part of clinicians and caregivers, and increase self-acceptance and optimism about recovery on the part of those affected.

## 1. Introduction

Anorexia nervosa (AN), bulimia nervosa (BN), and related eating disorders (EDs) have traditionally been viewed as “sociocultural creations,” or as products of disturbed family environments [1,2]. Not to downplay social and family influences in certain instances of ED, empirical evidence suggests that environmental “impacts” influence ED development by acting upon an environmentally malleable, heritable biology. In this paper, we review evidence for the concept that EDs involve the environmental regulation (through epigenetic processes) of hereditary susceptibilities, and discuss various potential benefits of providing patients, caregivers, and clinicians with a good understanding of a putative causal interplay, in the EDs, between genetic and environmental processes. Our intention with this paper is not to conduct a systematic review on epigenetics in the eating disorders (as such reviews exist elsewhere (e.g., [3,4]), but rather to provide an informed update on the latest findings, along with a discussion of clinical implications. Nonetheless, our review covers important recent articles related to the topic, as identified using PubMED and Google Scholar.

## 2. What are Eating Disorders (EDs)? 

Eating disorders (EDs) are characterized by intense preoccupations with eating, weight, and body image, and such maladaptive eating practices as excessive caloric restraint, binge eating, self-induced vomiting, and compulsive exercise [5]. These disorders are often associated with marked morbidity and mortality [6,7], and high personal and social costs, including lower educational and vocational achievement, decreased quality of life, and social isolation [7,8,9]. The current version of the Diagnostic and Statistical Manual of Mental Disorders (DSM-5) refers to EDs as “Feeding and Eating Disorders” (FEDs), and recognizes six subtypes: anorexia nervosa (AN), bulimia nervosa (BN), binge eating disorder (BED), avoidant/restrictive food intake disorder (ARFID), rumination disorder (RD), and pica. Two residual diagnoses (other specified feeding or eating disorder and unspecified feeding or eating disorder) capture ED variants that have clinical significance without fulfilling criteria for full-threshold syndromes [5]. 

AN is defined by restriction of energy intake and persistent avoidance of weight gain, resulting in subnormal body weight. The disorder has two sub-types: AN-restricting type, characterized by restriction of food intake without binging or purging; and AN-binge-eating/purging type, in which individuals regularly engage in binge-eating followed by compensatory behaviors (like self-induced vomiting or misuse of laxatives). People with BN also display recurrent binge-eating and compensatory behaviors, but in the absence of marked weight loss [5]. Individuals with BED, roughly 40% of whom are obese [10], show recurrent binge-eating without compensatory behaviors [5]. Newly introduced in DSM-5, ARFID is characterized by restriction of food intake for reasons unrelated to weight and body image—for instance, compulsive avoidance of foods judged to be impure or unhealthy, or that elicit disgust or aversion [5]. People affected by RD bring already swallowed foods back up and re-chew, whereas pica is a disorder in which people eat non-food items [5]. To date, there are no genetic or epigenetic studies involving ARFID, RD, or pica and, thus, our review does not address these entities. 

## 3. Etiology

EDs are understood to be multiply determined by genetic factors (that shape emotion regulation, reward sensitivity, energy metabolism, appetite, and other variations), environmental triggers (including perinatal insults, developmental stressors, later-life stressors), state-related effects (owing to the nutritional and mental status) and ultimately, social inducements toward intensive caloric restraint [11,12]. 

### 3.1. Heredity

Multiple sources of evidence point to the importance of heredity in the EDs. Family aggregation studies have shown that AN and BN are substantially more common in female first-degree relatives of people who themselves have AN or BN (e.g., Strober, et al. [13]), and one study shows that levels of eating and body-image concerns correspond in biological sister pairs, but not in adoptive pairs [14]. Even more convincingly, studies comparing concordance rates for EDs between mono- and dizygotic twin pairs reveal heritability coefficients for AN, BN, and BED ranging from 32% to 76% [15], 28% to 83% [15], and 39% to 45% [15], respectively. Aside from emphasizing unique genetic effects, these studies generally indicate non-shared environmental factors (e.g., a particular stressor experienced by one twin) to be more influential in ED development than are shared environmental factors (e.g., living in the same family environment) [15]. The preceding tends to refute ED formulations that are couched strongly in terms of family dynamics.

### 3.2. Genetics 

There have been various efforts to identify gene variants that may be associated with risk of an ED. Candidate-gene approaches (which examine single gene variants) are generally driven by a theory-based “guess” about which of the tens of thousands of genes may be an important contributor to risk for a disorder—something akin to guessing around which star in the night sky might there be a life-harboring planet. Nonetheless, some findings point to associations between EDs and polymorphisms of genes regulating key neurotransmitters (e.g., serotonin [16]), neuromodulators (e.g., brain-derived neurotropic factor (BDNF) [17]), hormones (e.g., estrogen [18]), or eating behavior (e.g., ghrelin [19]). Despite some leads, the candidate approach has not yielded many replicable findings. Detailed reviews of findings from candidate-gene studies in EDs can be found in several previous reviews (e.g., [3,20]).

Recent technologies support genome-wide association studies (GWASs), which allow for hypothesis-free investigations aimed at uncovering novel genetic markers that reach what is called “genome-wide statistical significance” for association with a phenotype of interest. Given that genome-wide methods test thousands of common human gene variant markers at a time, a strict threshold for significance (*p* < 5 × 10^−8^) has been established to correct for chance associations that could occur when conducting large numbers of simultaneous comparisons. The cost of maintaining such stringency, however, is that genome-wide studies require enormous sample sizes.

To date, GWASs in the field have been completed only for AN—although studies examining BN and BED are underway. The earliest published GWASs in AN were underpowered, and hence failed to yield findings of genome-wide significance [21,22,23,24]. However, two recently published GWASs, both conducted by the Eating Disorders Work Group of the Psychiatric Genomics Consortium (PGC-ED), have yielded intriguing findings reaching genome-wide significance. The first of these, involving DNA from 3,495 people with AN and 10,982 normal-eater controls, associated a locus on chromosome 12 with AN, at a site linked to type-1 diabetes and autoimmune diseases [25]. In addition, genetic correlations (computed using linkage disequilibrium scores) associated AN with mental-illness phenotypes (e.g., neuroticism) and physical-health phenotypes (rapid glucose and lipid metabolism, low body mass index (BMI)). In other words, in addition to expected psychiatric components, findings characterized AN as having important metabolic and autoimmune components. The autoimmune aspect of these findings, incidentally, corroborates a report from a study in more than 930,000 Swedish hospital records showing that children having a parent with an auto-immune disorder are unusually likely to develop an ED [26]. 

The second GWAS, involving data from 16,992 people with AN and 55,525 controls [27], identified eight significant genetic loci, and again implicated psychiatric traits (e.g., obsessive-compulsive and major depressive disorders), metabolic traits (e.g., insulin resistance, lipid metabolism) and anthropometric traits (e.g., low BMI, low fat mass). According to these studies, the genetic architecture of AN not only implicates psychiatric traits, but also metabolic factors and particular physical (anthropometric) characteristics.

## 4. Genes and Environmental Activation

It is an intuitive point that the genetic contribution to many mental-health problems acts only when triggered by the environment—and the preceding is very likely to be true of EDs. As we have already noted, twin data have shown that EDs involve a strong genetic diathesis, but also a contribution from the non-shared environment [15]. In other words, ED development is likely to implicate gene-environment interaction effects. Supporting this point, animal data suggest that genetic susceptibility, in combination with adolescent social stress and caloric restraint can produce a mouse “analog” to AN—mice that let themselves starve [28]. Similarly, clinical data show risk of AN to be increased in genetically disposed individuals when subjected to familial distress [29]. Diverse environmental influences have been postulated to act in AN, including obstetric insults, gestational stress, childhood trauma, familial conflict, adult victimization experiences, social inducement towards caloric restraint, and one’s actual nutritional state [30].

### Epigenetic Processes 

Epigenetic processes influence gene expression (and corresponding phenotypic variations) in the absence of actual DNA sequence changes, and are believed to act in an environmentally responsive fashion [31,32]. Mechanisms involved include DNA methylation/demethylation and hydroxylmethylation, histone acetylation/deacetylation, histone phosphorylation/ dephosphorylation, noncoding RNA and microRNAs, as well as transcriptome actions [32]. The most widely studied of these, DNA methylation, involves the addition of methyl groups to regions of the gene referred to as CpG sites—at which cytocine is followed by guanine [32]. Methylation in certain CpGs can silence or suppress gene expression—in theory, allowing for environmental programming of gene expression [31,32]. Available findings provide compelling evidence of a role of DNA methylation in rendering gene expression responsive to environmental exposures. However, these findings are not without limitations—from both methodological and conceptual standpoints. As a full discussion of such limitations is beyond the scope of this paper, the interested reader is referred to full treatments of such questions presented elsewhere [4,33].

#### Environments of Concern

##### The Prenatal Environment

A famous study, capitalizing on a “natural experiment” in malnutrition, showed that Dutch children born to starving mothers (due to a World War II induced food blockade) showed altered physical stature and emotional adjustment and, after six decades, altered DNA methylation in genes regulating growth and metabolism compared to those of their siblings [34]. In a similar vein, studies have linked maternal depression during gestation to altered methylation of the glucocorticoid receptor (*NR3C1*) gene and altered stress reactivity in the offspring [35]. Likewise, Suarez et al. [36] observed that maternal antenatal depression was associated with an epigenetic marker (lower epigenetic gestational age) associated with mental-health problems in pre-school age boys. Fathers matter, too. Germ cells in the sperm line convey epigenetic information, meaning that fathers can also influence fetal programming in their offspring [37]. There is a now-sizable literature demonstrating that stress in parents of both sexes can impact neurodevelopment in their offspring via epigenetic processes (see [38] for a thorough review). Also of relevance are findings of a recent study associating fathers’ periconception BMI with DNA methylation patterns in their children at age 3 and 7 years, independently of mothers’ BMI [39].

One of our group’s recent studies showed that children of mothers who were exposed to intense, third-trimester gestational distress during very-severe weather conditions—the 1998 Quebec Ice Storm (regarded as Canada’s worst natural disaster)—showed more ED symptoms at age 13 ½ than did children of mothers who had less environmental-stress exposure [40]. Indicating the effect to have likely epigenetic origins, in a separate study, degree of *in utero* stress exposure in the same children was associated with extent of alteration of methylation in genes involved in Type-1 and -2 diabetes mellitus [41]. Interestingly, children who had higher methylation levels at age 13 concurrently had a lower BMI and lower central adiposity [41]. Investigating possible epigenetic effects of maternal EDs upon their offspring, Kazmi and colleagues [42] measured genome-wide methylation of cord blood DNA in 21 babies of women with active AN, 43 with a past AN, and 126 normal-eater controls. Infants of women with AN had lower global methylation levels than did controls. In addition, babies of women who were actively eating-disordered during pregnancy had altered methylation in genes implicated in biosynthesis of cholesterol and neuronal survival, whereas those whose mothers once had AN showed altered methylation in a gene linked to inflammation and immune response. 

##### The Childhood Environment

Although it is uncertain to what extent findings obtained in animal studies inform processes in humans, animal studies have one advantage when studying possible epigenetic effects of early-life stress—as environmental stress exposures can be randomized across animals and experimentally manipulated and controlled in a way that could never be done in studies on developing humans. The preceding allows for differentiation of gene-environment correlations (in which the actor, because of a particular trait, induces an environmental effect) from gene-environment interactions (in which the environment has an action that is fully independent of the actor). There are many demonstrations that animals raised in stressful conditions show altered DNA methylation in systems associated with stress accommodation (e.g., [43,44,45]. Studies by Roth and colleagues indicate that infant rats exposed to adverse rearing experiences evinced lasting changes in the methylation status of the *BDNF* gene, and corresponding behavioral alterations [45]. Likewise, studies by Meaney and colleagues showed that rat pups receiving low maternal care compared to pups, that received high maternal care had greater hippocampal DNA methylation of the glucocorticoid receptor gene (*NR3C1*) and decreased hippocampal glucocorticoid receptor messenger RNA (mRNA) expression (e.g., [43,46]). In parallel, in a postmortem study, human suicide victims who had experienced childhood abuse have been shown to display higher methylation of the *NR3C1* gene promoter and decreased levels of glucocorticoid receptor mRNA in the hippocampus relative to non-abused suicide victims and controls [44]. Numerous other observations in humans associate early-life stress exposures (such as physical or sexual abuse, deprivation from parental care, or natural disasters), with epigenetic alterations and later behavioral and mental-health outcomes [33,47,48,49,50,51].

Suggesting that comparable effects apply in EDs, we previously found that women with BN who report a history of suicidality have greater methylation of specific CpG sites in the *NR3C1* gene promoter region [52]. We also observed hypermethylation of specific CpG sites in the promoter region of the *BDNF* gene in bulimic women who report a history of childhood sexual or physical abuse [53]. BDNF is thought to play a role in neural plasticity, learning of traumatic memory and binge eating [54]. Furthermore, we reported that women with BN and comorbid borderline personality disorder, who tend to report high levels of childhood adversity, show hypermethylation of the dopamine *DRD2* receptor gene promoter region [55].

##### The Nutritional Environment

Since EDs profoundly affect nutritional status, an important question is “How do ED-induced nutritional deficits affect the epigenome?” An even more important question may be: “Does nutritional rehabilitation during ED recovery reverse disorder-linked epigenetic alterations?”. Central to the preceding questions is that nutrients, such as folate, B12, and choline, influence the functioning of one carbon metabolism—a physiological process crucial for generating methyl-transfer reactions upon which DNA methylation depends [56]. While most of the evidence for the influence of nutrients on DNA methylation comes from animal studies, there is now increasing evidence in humans that dietary intake of folate, choline, and B-vitamins also affect DNA methylation and brain function across the life-cycle [56,57]. Furthermore, numerous studies suggest that peoples’ nutritional state can make epigenetically mediated contributions to psychiatric disorders [58]. Our group is presently studying pathways, in people with AN, linking self-reported eating behaviors, plasma levels of nutrients involved in one-carbon metabolism, and DNA methylation levels. At the time of this writing, results are in a preliminary state. However, initial indices suggest that nutritional factors do impinge directly upon DNA methylation levels [59].

## 5. Findings on DNA Methylation in People with EDs 

### 5.1. Methylation Studies in Candidate Genes

We emphasize from the start that candidate-gene methylation studies are subject to all of the limitations inherent in any candidate study—including problems of power and stability of results. Nonetheless, interpreted judiciously, available studies offer some intriguing indications: Available studies in AN have reported altered methylation of genes regulating expression of alpha-synuclein (involved in neurotransmitter release) [60], dopamine [61] (implicated in mood, impulse-control, reward sensitivity and binge-like eating), oxytocin [62,63] (linked to social attachment), histone deacetylase [64] (broadly influencing gene expression), and leptin [65] (which inhibits hunger). Combining measures of serotonin transporter (*SLC6A4*) gene methylation with resting-state functional connectivity data, Boehm, et al. [66] associated epigenetic variation in the *SLC6A4* gene with neural connectivity in the salience network—an important brain circuit for emotion regulation. Another research group reported that individuals with BN had greater methylation of the atrial natriuretic peptide (ANP) gene promoter (involved in cardiovascular homeostasis) than controls [67]. Similarly, studies noted earlier have documented alterations in the methylation of candidate genes that might, in theory, be relevant to bulimic ED variants—including glucocorticoid receptor [52] and dopamine *DRD2* receptor genes [55]. However, we note that available findings seem to indicate that the epigenetic variations noted may be more pertinent to comorbidity (e.g., suicidality or personality disorders) than to BN itself.

We identified no studies investigating methylation differences between patients with a primary diagnosis of BED and normal-eater controls. However, one study reported that patients who showed concurrent bipolar disorders and binge-eating had hypomethylation of the *SLC1A2* gene (involved in removing glutamate from the synaptic cleft) relative to patients with bipolar disorder who displayed no binge eating [68].

### 5.2. Global Methylation Level Studies

A handful of recent studies has attempted to quantify global methylation across the whole genome. Such studies allow for the evaluation of hypotheses about global variations in methylation levels, but are “blind” to possibly more meaningful variations in methylation acting at specific genomic loci. Studies of this type have tended to produce inconsistent results, largely (we suspect) because of small samples and a diversity of methods involved. Across available studies comparing participants with AN to those with no ED, we find one reporting no global differences [69], two reporting global hypomethylation in AN [60,70], and one reporting hypermethylation [71].

### 5.3. Epigenome-Wide Methylation Studies

Superior to either candidate-gene or global methylation measures, genome-wide methylation measures allow for the analysis of site-specific alterations at multiple genomic loci. A first study of this kind, performed by our group, used a high-throughput (Illumina 450K) technology to perform a genome-wide comparison of methylation levels in DNA obtained from leukocytes in 30 women with active AN and 15 normal-weight, normal eaters [71]. False discovery rate corrected comparisons identified differentially methylated CpG probes corresponding to genes associated with histone acetylation, cholesterol storage, lipid transport, and dopamine and glutamate signaling. Findings also linked chronicity of illness to DNA methylation levels at probes that mapped onto genes associated with anxiety, immunity, and central nervous system functioning. An independent study (using the same technology) reported on methylation in 47 females with AN and 100 population-based control females [72]. Intriguingly, two of the differentially methylated genes identified in case-control comparisons—*NR1H3* (involved in lipid metabolism and inflammation) and *TNXB* (associated with connective-tissue disorders)—corresponded to those identified by our group. TNXB encodes an extracellular matrix glycoprotein, absence of which has been associated with Ehlers–Danlos syndrome, a connective tissue disorder characterized by joint hypermobility that is noted to co-occur with AN [73].

Our group subsequently reported on an expanded methylome-wide study, involving enlarged samples of participants with active AN or no ED, and a new sample of individuals in whom AN had remitted for at least one year [74]. Methylation levels in members of the remitted group differed from those in the active group on probes that, among others, isolated genes associated with serotonin and insulin activity, glucose metabolism, and immunity. Intriguingly, the direction of methylation effects in remitted participants tended to be opposite to that seen in individuals with active AN, suggesting that epigenetic alterations in actively ill individuals may be reversible. If so, DNA methylation could serve as a marker of disease staging or therapeutic response. Furthermore, we find it intriguing that altered methylation findings seem to parallel results of the GWASs described earlier [25]—i.e., they also implicate psychiatric, metabolic, and immune functions.

## 6. Clinical Applications

Traditional etiological models are replete with blameful and pathologizing innuendos concerning roles in ED development of “maladaptive personality traits” in affected individuals and “problematic interaction patterns” in their families. As our review has shown, contemporary conceptualizations refute such notions—promoting instead the point that people do not develop EDs because of “character weaknesses,” “stubbornness,” “superficial concern with appearance,” or “bad parenting” but because they carry real genetic susceptibilities that get “switched on” by a lifetime of environmental exposures. In other words, EDs are understood to result from factors that are far beyond the willful control of those affected. It is our belief that perspectives on ED development that properly accommodate genetic and epigenetic influences improve clinicians’ sensitivity to their patients’ realities, and help make treatment more palatable and humane. 

We take inspiration from several recent efforts to apply gene x environment interaction concepts clinically in the ED area. One recent study showed that an approach to ED psychoeducation that was couched in epigenetic terms (referred to as “malleable biology”), when compared to approaches framed in purely biological or cognitive terms, led to greater recovery optimism and felt self-efficacy on ED patients’ part [75]. A recent paper makes a similar point, that when counseling for people affected by EDs places causal responsibility upon interacting genetic and environmental influences, it has potential to relieve blame and to legitimize patients’ experiences [76]. One of the co-authors of the paper in question, Jehannine Austin, has been a strong proponent of the application, across a variety of mental-health contexts, of a new “breed” of genetic counseling. She and her colleagues have shown that counseling that informs patients and those close to them about gene-environment interactions helps empower and increase self-efficacy in individuals with mental illness [77]. In one of their studies, patients reported that this style of counseling made them better able to manage their illnesses, and more open to talking about them with family and friends [77]. We have similarly argued that epigenetically informed models improve clinicians’ empathy surrounding the ways in which EDs can become entrenched and difficult to overcome [30]. Encouragingly (and a bit paradoxically), formulations of ED development that accommodate neuroscience concepts actually seem to “humanize” the understanding of ED illness and recovery. Arguably, genetically and epigenetically informed models of ED development contribute positively to efforts of clinicians and carers in various ways:

1. They blame affected individuals less. Since the causes of EDs are increasingly understood to involve the activation of real physical susceptibilities by real environmental exposures, it becomes possible to trace with patients the sequence of life events (that may include perinatal insults, childhood adversities, school-related stresses and, invariably, the effects of prolonged caloric restraint) that served to activate inherited susceptibilities toward ED development. Likewise, because they take into account multiple causal factors (and complex interactions among them), informed models do less “finger pointing” at parents and other caregivers. It is never a single event or action (e.g., parents’ divorce, or a care-taker’s depressive episode) that caused someone’s ED.
2. They help promote greater self-acceptance. Clinical experience dictates that a common “symptom” of an ED is shame. People invariably feel stupid to have developed their disorder, weak to not yet have overcome it, and guilty for the distress their disorder causes relatives and friends. When with someone experiencing shame around his/her ED, and speaking from an epigenetically informed understanding, we might often say something like: “You didn’t ask to have an ED. At the end of the day, when you fully understand why you developed this disorder, you won’t have to feel ashamed. You’ll just say, ‘I see why I got an ED’”. This stance on therapists’ part, when sincere, helps promote self-acceptance in people who are prone instead to self-disparagement. Likewise, especially when afflicted by an ED after several rounds of therapy, or decades of suffering, it is natural for affected people to feel inadequate, and perhaps deserving of messages they may have received from uninformed carers or therapists that “you aren’t trying hard enough” or “You’re choosing to keep your ED”. Findings from the epigenetic literature suggest that chronic exposure to malnutrition and dietary distress amplify psychological tendencies (e.g., compulsivity, anxiety) and metabolic adaptations (e.g., altered lipid metabolism) that help “lock” the ED into place. The difficulty one may experience in recovering from an ED becomes understood, not as an index of character weakness or obstinacy, but of the extent to which biological processes anchor symptoms and behaviors into place.
3. They help patients (and therapists) accept “incremental response.” Epigenetic data in AN suggest that there are many disorder-induced alterations in the expression of genes that affect mental status, metabolism, and immune/inflammatory processes—and that such alterations become more pronounced with increasing chronicity of illness (see [71,74]). It is likely that these same factors need to be “reset” before someone affected can take back control. Encouragingly, some findings show that nutritional rehabilitation does help undo problematic changes—but it remains unclear over what span of time such alterations take place.
4. They assign proper importance to nutritional factors. It is clear that malnutrition and dietary distress amplify physical and psychological problems in ED patients, and help lock the disorder into place. Various recent findings suggest that epigenetic processes may contribute to ED entrenchment through nutritionally-induced alterations in gene expression [71,72,74]. An implicit message is that: ”Your ED was triggered by too much caloric restraint and, logically, recovery will depend upon re-establishing a healthy nutritional state”. Although further research is required to establish parameters, a related concern may help moderate messages aimed at preventing obesity that encourage dietary restraint.
5. They help separate the person affected from his/her illness. “Externalizing the illness” is an explicit operation in family-based treatment approaches [78], and an implicit one in Cognitive Behavioral Therapy [79] and other established ED treatments. Recognizing that one is separate from one’s disorder (and the behaviors that it drives) helps affected people overcome shame, and increases empathy on the part of family members, partners, and friends. A genetically/epigenetically informed model implicitly separates individuals from the factors that caused and perpetuate their illnesses—in the sense that the model makes explicit the point that EDs represent the activation of heritable physical susceptibilities by real-life experiences. We often say: “You did not ask to have this disorder. You are responsible for repairing the damage and recovering, but not for what caused the illness in the first place”. In a related vein, because of its ego-syntonic nature, people with AN sometimes identify positively with their disorder, or assume it as an identity. We believe that an epigenetically informed perspective helps counteract such tendencies. It helps people affected by the disorder recognize that “you are not ‘an anorexic’. Rather, you are someone in whom a vulnerability has been switched on by too much dieting. And the effect is that restricting food intake feels good in a bit the same way that abusing drugs feels good to a person with an addiction.”

The following clinical vignette illustrates the potential value of an epigenetically informed stance: Some time ago, we admitted a woman with severe anorexia nervosa onto our inpatient unit. She was bewildered and frightened in the early days of her stay on our unit. A voluntary patient, after only a few days she took essentially the stance: “Thank you, I’m feeling much better and would like to go home.” This was not a plan that could be safe for her. As a program invested in practices governed by notions of autonomy support (See [80,81]), we made efforts to avoid involuntary admission. But rational arguments aimed at helping this woman opt to stay voluntarily failed to be convincing, and she became increasingly adamant about returning home. Thankfully, serendipity made involuntary admission unnecessary. This woman spontaneously asked why she felt so bloated after eating, and we explained that this was a symptom of delayed stomach emptying time typical in AN. She replied: “You mean it’s not in my head”. We replied: “No. It’s not in your head; it’s in your stomach. There’s a real physical cause”. We explained further. “And speaking of real physical causes, you know that study on epigenetics in which we asked you to take part? Can we talk about that a bit?” We elaborated on what epigenetics is, how it is a science that promises to explain how genetic tendencies get activated by the environment. How life stresses and too much dieting seem to be among the factors that cause epigenetic changes in AN. After a brief discussion, and after addressing the patient’s various questions and confusions, she stated: “That’s so interesting”. Signs of indignity fell away. She not only consented to take part in the study, but agreed to stay longer on the unit. 
